# *Announcement: *World No Tobacco Day — May 31, 2017

**DOI:** 10.15585/mmwr.mm6620a7

**Published:** 2017-05-26

Each year, the global tobacco epidemic kills an estimated 6 million persons worldwide, including 600,000 who die from secondhand smoke exposure. If current trends continue, it is estimated that by 2030 tobacco use will result in approximately 8 million deaths worldwide annually; an estimated 80% of these preventable deaths will occur in low- and middle-income countries (*1*).

World No Tobacco Day, sponsored by the World Health Organization (WHO) and observed on May 31 each year, highlights the health risks associated with tobacco use and encourages effective actions to reduce tobacco consumption. This year, the theme for World No Tobacco Day is “Tobacco — a Threat to Development” (*2*).

To support this theme, WHO is calling for activities that include international collaboration highlighting the links between the use of tobacco products, tobacco control, and sustainable development. In addition, WHO is calling for activities that demonstrate ways that individuals can contribute to bringing about a sustainable, tobacco-free world, either by committing to never start using tobacco products or by quitting such use (*2*).
